# Dietary-Induced Elevations of Triglyceride-Rich Lipoproteins Promote Atherosclerosis in the Low-Density Lipoprotein Receptor Knockout Syrian Golden Hamster

**DOI:** 10.3389/fcvm.2021.738060

**Published:** 2021-11-02

**Authors:** Xiao Lin, Ping Ma, Chun Yang, Jinjie Wang, Kunxiang He, Gonglie Chen, Wei Huang, Jianglin Fan, Xunde Xian, Yuhui Wang, George Liu

**Affiliations:** ^1^Key Laboratory of Molecular Cardiovascular Science, Ministry of Education, Institute of Cardiovascular Sciences, School of Basic Medical Sciences, Health Science Center, Peking University, Beijing, China; ^2^Department of Molecular Pathology, Graduate School of Medicine, University of Yamanashi, Chuo, Japan

**Keywords:** triglyceride-rich lipoproteins, ezetimibe, atherosclerosis, low-density lipoprotein receptor, Syrian golden hamster

## Abstract

Elevated triglycerides are associated with an increased risk of cardiovascular disease (CVD). Therefore, it is very important to understand the metabolism of triglyceride-rich lipoproteins (TRLs) and their atherogenic role in animal models. Using low-density lipoprotein receptor knockout (LDLR^−/−^) Syrian golden hamsters, this study showed that unlike LDLR^−/−^ mice, when LDLR^−/−^ hamsters were fed a high cholesterol high-fat diet (HFD), they had very high plasma levels of triglycerides and cholesterol. We found that LDLR^−/−^ hamsters exhibited increased serum TRLs and the ApoB100 and 48 in these particles after being fed with HFD. Treatment with ezetimibe for 2 weeks decreased these large particles but not the LDL. In addition, ezetimibe simultaneously reduced ApoB48 and ApoE in plasma and TRLs. The expression of LRP1 did not change in the liver. These findings suggested that the significantly reduced large particles were mainly chylomicron remnants, and further, the remnants were mainly cleared by the LDL receptor in hamsters. After 40 days on an HFD, LDLR^−/−^ hamsters had accelerated aortic atherosclerosis, accompanied by severe fatty liver, and ezetimibe treatment reduced the consequences of hyperlipidemia. Compared with the serum from LDLR^−/−^ hamsters, that from ezetimibe-treated LDLR^−/−^ hamsters decreased the expression of vascular adhesion factors in vascular endothelial cells and lipid uptake by macrophages. Our results suggested that in the LDLR^−/−^ hamster model, intestinally-derived lipoprotein remnants are highly atherogenic and the inflammatory response of the endothelium and foam cells from macrophages triggered atherosclerosis. The LDL receptor might be very important for chylomicrons remnant clearance in the Syrian golden hamster, and this may not be compensated by another pathway. We suggest that the LDLR^−/−^ hamster is a good model for the study of TRLs-related diseases as it mimics more complex hyperlipidemia.

## Introduction

Cardiovascular diseases are a leading cause of death and are associated with metabolic syndromes including hyperlipidemia, non-alcohol fatty liver, diabetes, hypertension, and obesity (1, 2). Atherosclerosis is the underlying pathology of many cardiovascular diseases (CVD), while hyperlipidemia is the most common contributing factor. So far, animal models of atherosclerosis have demonstrated that plaque formation begins with the infiltration and oxidation of low-density lipoproteins (LDL) in the arterial wall (3). However, the role of triglyceride-rich lipoproteins (TRL) is poorly understood, and hence, the role of TRLs in atherosclerosis needs to be further investigated (4). While it has previously been demonstrated that severe hypertriglyceridemia promoted atherosclerosis in lipoprotein lipase knockout (LPL^−/−^) mice, disease progression was very slow, taking more than 1 year to detect atherosclerosis (5). It is possible that very large lipoprotein particles in LPL-deficient animal models have difficulty entering the arterial wall. Further, different types of hypertriglyceridemia detected in clinical settings suggest that atherogenic mechanisms are more complex than previously thought. Therefore, a more appropriate animal model is required to investigate the role of TRLs in atherosclerosis.

The Syrian golden hamster is widely used to study lipid metabolism. This is because hamsters and humans have comparable lipid metabolisms, including that of cholesteryl ester transfer protein (CETP) activity and the “LDL-based” lipoprotein profile in the blood (6–8). Previous studies have shown that, unlike in mice and rats, blood lipid levels in wild type (WT) hamsters were regulated by lipids and fructose in the diet, where blood triglyceride levels increased significantly (9, 10). Interestingly, it has been shown that the characteristics of postprandial hyperlipidemia were more evident in LDL receptor knockout (LDLR^−/−^) hamsters (11–14). Further, lipid-lowering drugs had differing effects on hamsters to mice and rats. Inhibition of cholesterol synthesis by statins was very toxic in the hamster, especially when fed a cholesterol-rich diet (11, 15, 16). Moreover, inhibition of cholesterol absorption by ezetimibe completely reversed the increased plasma lipid levels in hamsters fed atherogenic diets, and this effect was not altered by a compensatory increase in cholesterol synthesis (9, 17, 18).

Using the LDLR^−/−^ hamster, this study investigated lipid metabolism and atherosclerosis in mixed hypercholesterolemia and severe hypertriglyceridemia. As previous reports have shown that hamster plasma lipid levels were affected by cholesterol from the intestine, this study aimed to assess the lipid profile of LDLR^−/−^ hamsters when fed high cholesterol high fat (simply termed high-fat diet HFD for ease) diets either with or without ezetimibe treatment. Parallel comparison of LDLR^−/−^ hamster and mouse responses to ezetimibe have not been reported, as well as the comparison of LDLR^−/−^ hamster responses between chow diet (CD) and HFD. Because of the cholesterol absorption inhibitory effect of ezetimibe, this study sought to determine the characteristics of the hyperlipidemia LDLR^−/−^ hamster animal model and evaluate the effects of intestinally-derived cholesterol on a range of lipoproteins and atherogenesis. Through this, it was hoped that the analysis of lipid metabolism and its influence on the disease would determine the appropriateness of using the LDLR^−/−^ hamster for such studies in the future.

## Materials and Methods

### Animals

Syrian golden hamsters and C57BL/6 mice were purchased from Beijing Vital River Laboratory Animal Technology Co., Ltd. (Beijing, China). LDLR^−/−^ hamsters were generated by CRISPR/Cas9 genetic editing system in our lab and bred at the SPF animal facility of Hebei Ex&Invivo Biotechnology Co. (Hebei, China) as described previously (9). LDLR^−/−^ mice were provided by the genetically modified animal model platform of the Key Laboratory of Molecular Cardiovascular Sciences of the National Science and Technology Ministry (Beijing, China). All animals were fed with a normal CD (20% protein and 4% fat; purchased from Beijing Ke'ao Company, Beijing, China) or a high-fat diet (HFD) [0.5% cholesterol, 15% lard (w/w) based on CD] and water *ad libitum*. All animals were kept in a temperature-controlled environment on a light-dark cycle of 12 light/12 dark. The experimental procedures were handled according to the guidelines of the laboratory animal care (NIH publication no. 85Y23, revised 1996) and approved by the Animal Care and Use Committee of the Peking University Health Science Center (LA2015-012).

Considering the life span of the two kinds of rodents, we compared the mid-life animals in the experiments. As we have known, mice are generally considered to live for 1 year. According to the report (19) and our observation, the overall average life span of hamsters is 2 years. Therefore, female Syrian golden hamsters at 12 months of age, and 6-month-old female C57BL/6 mice were used in this study in order to induce atherosclerotic lesions more quickly. Thirty LDLR^−/−^ hamsters were divided into four groups: CD, *n* = 5; CD and ezetimibe (CD + EZE), *n* = 5; HFD, *n* = 10; HFD and ezetimibe (HFD + EZE), *n* = 10. Then, 20 WT hamsters were used as controls and were divided into four groups (see above) where for each group, *n* = 5. The WT or LDLR^−/−^ mice were divided into HFD and HFD+EZE groups (*n* = 10) as controls for the investigations involving the lipid-lowering treatment (ezetimibe) in hamsters. Ezetimibe was administrated by gavage dissolved in saline, and saline alone was the control. The dose of ezetimibe for hamsters was 2 mg/kg/day and for mice was 3 mg/kg/day, which is the equivalent dose for hamsters and mice based on body surface area. Hamster plasma was collected 1- and 2-weeks post-treatment for analysis of plasma lipid levels and lipoprotein profiles. After 40 days, hamsters were anesthetized with sodium pentobarbital and euthanized for tissue harvest.

### Plasma Lipid Analysis

Experimental animals were fed an HFD for 2 weeks, then animals fasted overnight and anticoagulated blood was collected and centrifuged (4,000 rpm, 4°C, 10 min) to separate plasma. The total cholesterol and triglyceride content was measured using commercially available assays from Biosino Biotechnology and Science, Inc. (Beijing, China). Fractions of plasma lipoproteins were separated and collected using an ÄKTA fast protein liquid chromatography (FPLC) system (Amersham Biosciences, USA). Pooled plasma (100 μl) from 5 aliquots per group were eluted with buffer at a constant flow rate of 1 ml/min. For each fraction, 500 μl eluate was collected for triglyceride and cholesterol concentration measurements. A unique feature of the HFD-fed LDLR^−/−^ hamster was the presence of high plasma concentrations of chylous, discernable upon visual inspection, which had not been previously observed in other HFD animal models in this study. The plasma lipoproteins from the HFD-fed LDLR^−/−^ hamsters were too large to be processed with a column because the column (superpose 6 HR10/30, GE) would be damaged. Pooled plasma was then centrifuged (15,000 rpm, 30 min) and the lower phase was used for loading to remove very large particles, which accounted for ~68% of triglyceride and 45% of the cholesterol of the estimated lipid content in the pre-treatment samples. The concentration of triglycerides and cholesterols in total plasma and the lower phase was determined, respectively, to calculate the number of large particles removed.

### Cell Culture

Human umbilical vein endothelial cells (HUVEC, 8000, ScienCell, USA) were maintained in an endothelial cell medium (1001, ScienCell, USA) at 37°C, 5% CO_2_ environment. Cells were subcloned into 6-well plates and equilibrated with 0.2% BSA-ECM for 6 h. Then, 5% (w/w) serum, which was separated from CD treated, HFD treated, and HFD with ezetimibe treated hamsters, was added into the cells for a further 24 h. Culture mediums were discarded and cells were washed 3 times with PBS. Protein was extracted by RIPA Lysis Buffer (R0020, Solarbio, Beijing, China) to prepare samples for subsequent western blot analysis.

### SDS-Page and Western Blot Analysis

Triglyceride-rich lipoproteins were separated by ultracentrifugation at 42,000 rpm for 18 h from 5 ml plasma of WT or LDLR^−/−^ hamsters with Optima XPN-100 Ultracentrifuge (Beckman Coulter, USA). Then, TRL samples with equal triglyceride concentrations were delipidated with an organic solvent and used for sodium dodecyl sulfate-polyacrylamide gel electrophoresis (SDS-PAGE) analysis. The gel was dyed with Coomassie Brilliant Blue to show apolipoprotein bands. For western blot analysis, 1 μl plasma (animal experiments) or 20 μg protein (cell experiments) were prepared with an SDS and dithiothreitol (DTT) buffer by heating at 95°C for 10 min. The samples were loaded to 10 or 6% SDS-PAGE gels and underwent electrophoresis at 110 V. Proteins were transferred onto nitrocellulose membranes for 60–180 min at 220 mA. Membranes were then blocked with 5% BSA, and hybridized with the following antibodies: anti-ApoB, anti-ApoE, or anti-ApoAI (ab20737, ab20874, ab20453 rabbit polyclonal IgG respectively, Abcam, U.K) for plasma analysis, or anti-ICAM-1 or anti-VCAM-1 (CST 4915s or 13662 respectively, Cell Signaling Technology) for cell lysate analysis. Because the same volumes of plasma were used for electrophoresis, no internal reference was required as the sample loading control. The anti-GAPDH (ab8245, Abcam, U.K) antibody was used for the internal reference and the loading control for cell lysate analysis. Target proteins were visualized upon incubation with horseradish peroxidase-conjugated secondary antibodies followed by enhanced chemiluminescence detection (Molecular Imager Gel Doc XR System, Bio-Rad, Hercules, CA, USA).

### Pathology Analysis

Tissues were fixed in 4% paraformaldehyde for 24 h and equilibrated in 20% sucrose for 24 h. For oil red O (Sigma-Aldrich, St. Louis, MO, USA) staining, heart and liver were embedded in optical coherence tomography (OCT), frozen at an approximate temperature of −20°C, and sectioned at 7 μm with a freezing microtome (Leica, Switzerland). The quantitative analysis of atherosclerosis was represented as the percentage of *en face* lesion area ratio to the whole area of the full-length aorta, and the total area of the aortic root lesion by image J software. For H&E, sirius red, and immunohistochemical staining, the tissue samples from the aortic arch for all hamster samples after *en face* analysis were embedded in paraffin and sectioned at 3 μm with a Leica microtome, following a standard protocol referring to our pathology platform. Immunohistochemical staining was performed with anti-VCAM-1 and anti-α-SMA antibodies (BA3840 and A03744 rabbit polyclonal IgG respectively, Boster, USA).

### Tissue Lipid Analysis

Lipid extraction was referred to as a modified method by Bligh and Dyer (20). Briefly, 100 mg of tissue was homogenized in 1 ml cold PBS. Then, lipids were extracted in 5 ml glass tubes to avoid polymer contamination by vortex with the same volume of chloroform/methanol (v:v = 2:1) for 90 s and then centrifuged at 2,000 rpm for 20 min. The chloroform layer at the lower phase was transferred using a glass syringe and the rest was repeated as above. The collected chloroform layer was dried under nitrogen. Lipids were dissolved with 3% TritonX-100 (T8200, Solarbio, Beijing, China) for analysis. The cholesterol and triglyceride content were measured with the kits described above.

### Quantitative Real-Time PCR Assay

Total RNAs were extracted with Trizol reagent (12183555, Invitrogen, USA) and 50 μg RNA was RT by a commercial RT kit (18091200, Invitrogen, USA). Real-time polymerase chain reaction (PCR) was performed using the AriaMx Real-Time PCR System with Top Green PCR Master Mix (AQ131-01, TransGen Biotech, USA). The primers used in real-time PCR are listed in Table 1 and GAPDH was used as the internal reference.

**Table 1:** The primers used in real-time polymerase chain reaction (PCR).

**Gene**	**Forward**	**Reverse**
ABCG5	CCATTCTGACTTACGGAGAGTTG	CAGGGGTAACCACAGTTATTGAA
ApoB	GTGTACGGCTTCAACCCTGA	TCAGGAATGGCCAGCTTGAG
CETP	TCCATAAGCTGCTCCTGCAC	GCCCTTGTGATGGGACTCAA
GAPDH	GACTCATGACCACAGTCCATGC	AGAGGCAGGGATGATGTTCTG
HMGCoA Synthetase	TCCTCCGTGGATTGCTCCTT	TCGGTCACTGTCTCCACCTT
HMGCoA Reductase	TGATGGGAGCTTGCTGTGAG	ACCAAGACCTATTGCCCTGC
MTP	AGAGGAAAACCTGGACTCCTATG	AGCATTTTGGACATCAGATCACT
NPC1L1	ATGGCCACTCACTGTCTTGG	CGTCGTGGAAAGCCTTCTCT
SRB1	TGCCCGTCATCTACCAGTTG	TTTGGGACCCTACAGCTTGG
SREBP1c	GCGGACGCAGTCTGGG	ATGAGCTGGAGCATGTCTTCAAA
VLDLR	GCCTACCAGCACCACAGATT	GGTCACATTGATCCTTTGACACT

### Quantification and Statistical Analysis

All data were expressed as the mean ± SEM, and statistical tests are specified in figure legends. GraphPad Prism 7 software (GraphPad Software, La Jolla, CA, USA) was used for all statistical analyses. For a comparison between two groups, the Mann-Whitney test was used. For comparisons between three or more groups, one-way ANOVA was used. *P* < 0.05 were considered statistically significant.

## Result

### Dietary Cholesterol Increased Triglyceride-Rich Lipoproteins in LDLR^–/–^ Hamsters

**Figure 1:**
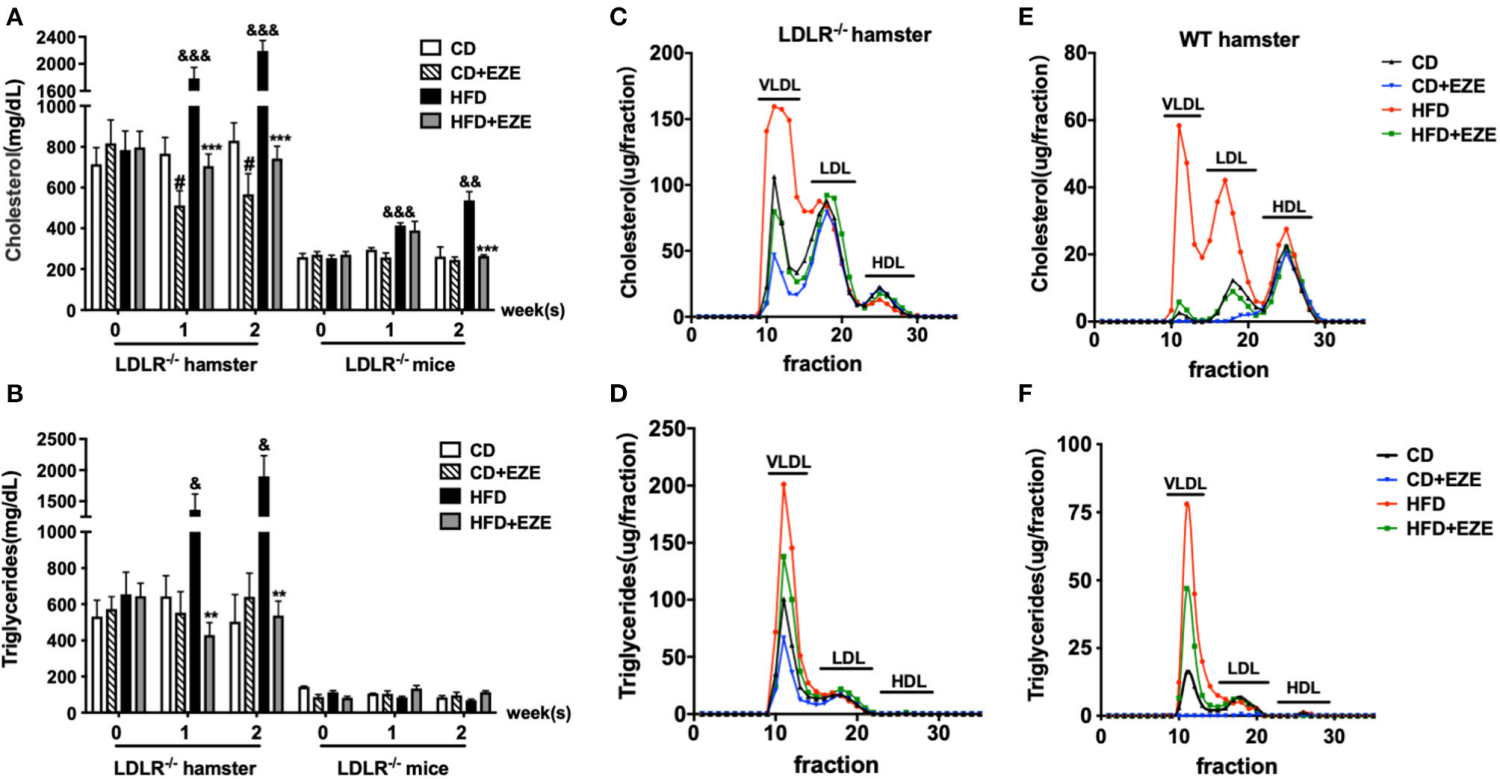
The responses to high-fat diet (HFD) and ezetimibe (EZE) in hamsters and mice. Total cholesterol **(A)** and triglyceride **(B)** levels were determined from plasma collected after 2 weeks of HFD and EZE treatment from LDLR^−/−^ hamsters (*n* = 10) and mice (*n* = 5). Data are shown as mean ± SEM. ^#^*p* < 0.05 CD+EZE vs. CD; ^&^*p* < 0.05, ^&&^*p* < 0.01, ^&&&^*p* < 0.001 CD vs. HFD; ^**^p < 0.01, ^***^p < 0.001 HFD + EZE vs. HFD, where a two-way ANOVA with Tukey's multiple comparisons test was performed. Lipid profiles of LDLR^−/−^ **(C,D)** and WT **(E,F)** hamsters. The plasma lipoprotein profile of hamsters 2 weeks after different treatments (CD, CD ± EZE, HFD, HFD ± EZE) was analyzed by FPLC. Each sample was of pooled plasma of five hamsters in each group, and 100 μL of the sample was loaded into the column. The cholesterol (upper) and triglycerides (bottom) of every collected fraction were determined and each data point was connected to make a curve on the graph. Each peak represents chylomicrons/VLDL, LDL, HDL fractions, respectively, from left to right.

As we have previously reported, hamster plasma triglyceride levels were sensitive to dietary cholesterol (9–11, 14, 21). In this study, we found that LDLR^−/−^ hamsters had significantly higher total cholesterol and triglyceride levels in the plasma when fed with CD, compared with LDLR^−/−^ mice (Figures 1A,B). Interestingly, an HFD diet dramatically increased plasma lipids levels in LDLR^−/−^ hamsters, especially triglyceride levels. In addition, in WT hamsters, an HFD also significantly increased the level of plasma cholesterol and triglycerides (Supplementary Figure 1A). Thus, whether the LDL receptor was present or knocked out, HFD-feeding in hamsters resulted in these marked changes. Therefore, we concluded that compared with other rodents, the hamster is special for studying lipid metabolism and related diseases with their obvious phenotypes.

After ezetimibe treatment for 2 weeks, there was a 30% decrease in total plasma cholesterol levels in CD-fed LDLR^−/−^ hamsters. Ezetimibe had no significant effect on plasma lipids levels in CD-fed WT hamsters or LDLR^−/−^ mice (Supplementary Figure 1A and Figures 1A,B). Ezetimibe completely prevented HFD-induced elevation of both cholesterol and triglycerides in hamsters and mice. Without ezetimibe treatment, plasma cholesterol and triglyceride concentrations were more than 3 times greater in hamsters up to more than 2,000 mg/dl. But in mice, HFD can only induce elevation of cholesterol, from about 200–500 mg/dl. But there was no significant increase of triglyceride concentration after HFD-induced in mice.

We also analyzed plasma lipoprotein profiles by FPLC to investigate which lipoproteins changed in response to diet and ezetimibe. The results showed that in LDLR^−/−^ hamsters, HFD-induced cholesterol changes occurred predominantly in the TRL fraction. The effect of ezetimibe was also restricted to the cholesterol in the TRL fraction (Figures 1C,D). In contrast, in WT hamsters, changes in plasma cholesterol in response to an HFD were observed in both TRL and LDL particles (Figures 1E,F). Increased triglycerides in TRLs indicated an increase in the number of these particles (Figures 1D,F).

This data suggested that in LDLR^−/−^ hamsters, nutritional, not endogenous, cholesterol contributed to the increase in blood lipids. In LDLR^−/−^ hamsters, it is the TRL fraction that was regulated by intestinally-derived cholesterol, but in WT hamsters, both TRL and LDL fractions were regulated. It was also evident that intestinally-absorbed cholesterol may be cleared by the LDL receptor in hamsters. Together, these current and previous (8, 21) results showed that in rodents, Syrian golden hamsters were more sensitive to changes in dietary lipids and more susceptible to hypertriglyceridemia. The LDL receptor might play an important role in this process. The LDL receptor was mainly involved in the clearance of triglycerides in plasma, and not in VLDL secretion (Supplementary Figure 2).

### Ezetimibe Prevented HFD-Induced Increase of Plasma ApoB48 and ApoE

**Figure 2:**
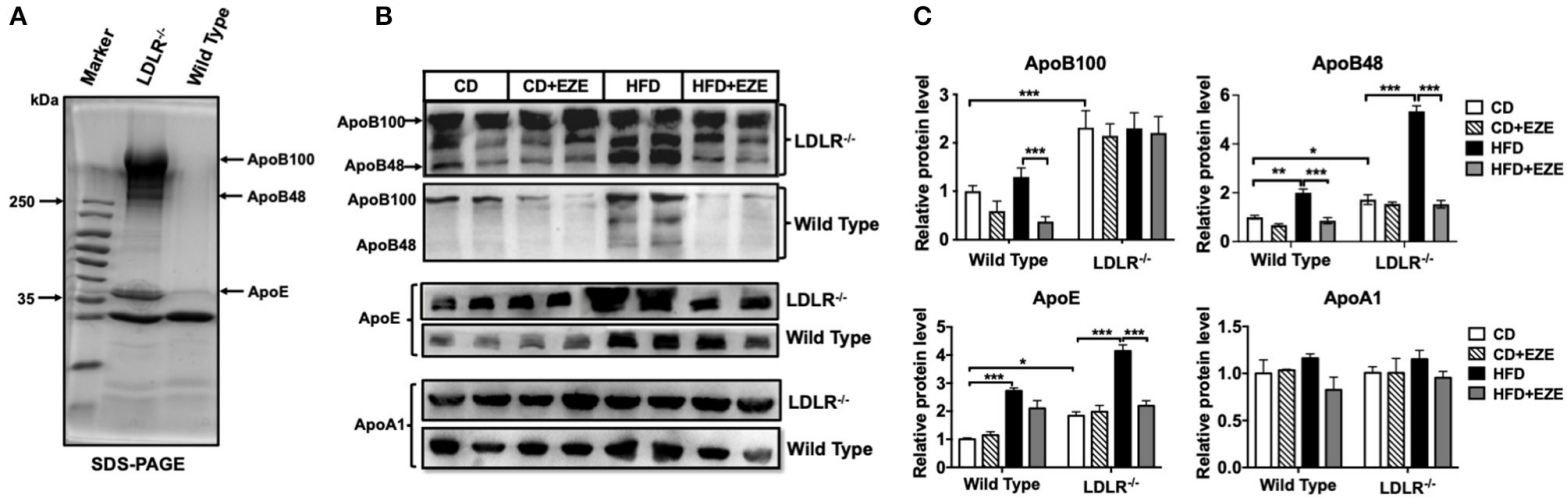
Apolipoproteins of TRLs were analyzed by SDS-PAGE **(A)** and western blotting **(B)** for ApoB, ApoE, and ApoAI in plasma of LDLR^−/−^ and WT hamsters with or without HFD and ezetimibe treatment after 2 weeks. **(C)** Is the quantitative bar chart analysis for **(B)**, the relative protein level was ratio to the CD group of WT hamsters. Separated TRLs from the plasma of fasted hamsters fed a CD were used for SDS-PAGE analysis. For each sample, 1 μL of plasma was used for analysis. Data are shown as mean ± SEM. Three-way ANOVA with Tukey's multiple comparison test, ^*^, ^**^, ^***^ represent *p* < 0.05, 0.01, and 0.001, respectively.

In order to better understand the different plasma lipoprotein profiles, in particular the apolipoproteins, in LDLR^−/−^ and WT hamsters, TRLs were analyzed using SDS-PAGE and 1 μl plasma was analyzed using western blot, respectively. Consistent with our findings on lipid concentrations and lipoprotein fractions, ApoB and ApoE in TRLs from LDLR^−/−^ hamsters increased compared with WT hamsters (Figure 2A). In LDLR^−/−^ hamsters, HFD led to a marked increase in both ApoB48 and ApoE in plasma, with no significant changes in ApoB100 and ApoAI, and ezetimibe largely prevented the increase of ApoB48 and ApoE (Figures 2B,C). However, in WT hamsters, ezetimibe treatment decreased ApoB100 levels both in CD and HFD, in addition, a decrease of ApoE after HFD. As known in the Syrian golden hamster, ApoB100 and ApoB48 are derived from liver and intestinal synthesis, respectively. These results further supported the notion that the dietary cholesterol increased TRLs in LDLR^−/−^ hamsters originated from an intestinal source. The changes in hamster lipoproteins very much resemble postprandial hyperlipidemia in humans. On the other hand, it suggested that the clearance capacity of chylomicrons and their remnants might be poor in the hamster, especially when the LDL receptor is deficient. Consequently, the LDL receptor likely plays an important role in the metabolism of these lipoproteins.

In addition, we examined ApoB100 and 48 contents in separated TRLs and FPLC TRLs' fractions of LDLR^−/−^ hamsters (Supplementary Figures 4A,B). From the results, the ApoB100 and 48 both increased in TRLs in the HFD group, and the ratio of ApoB100:48 was consistent in TRLs separated by ultracentrifuge and FPLC (about 1:1-1.3). However, ezetimibe can reverse this increase in TRLs fractions. Analysis by SDS-PAGE, ApoA5, and ApoC3 increased in the HFD group, while the ApoC2 did not change (Supplementary Figure 4C). These results also suggested that increased TRLs in HFD-fed hamsters were intestinally derived.

### TRLs Promoted Early-Stage Atherosclerosis Due to High LDL Levels

Most of the previous atherosclerosis studies were based on mouse and rabbit models which do not have severe hypertriglyceridemia (21–23). In this study, LDLR^−/−^ hamsters had severe hypertriglyceridemia and diet-affected lipoproteins allowed us to investigate the atherogenic properties of TRL particles. As the results indicated thus far, ezetimibe affected large lipoprotein particles in LDLR^−/−^ hamsters. Following this, we compared atherosclerosis with or without ezetimibe treatment to evaluate the role of TRLs. Our previous study showed that spontaneous atherosclerosis occurred in LDLR^−/−^ hamsters that were older than 15 months. Here, we were able to accelerate atherogenesis and successfully induce early atherosclerosis in 12-month-old LDLR^−/−^ hamsters, after 40 days of HFD. The levels of total cholesterol and triglycerides exceeded 3,000 mg/dl in HFD-fed LDLR^−/−^ hamsters (Supplementary Figure 1C).

**Figure 3:**
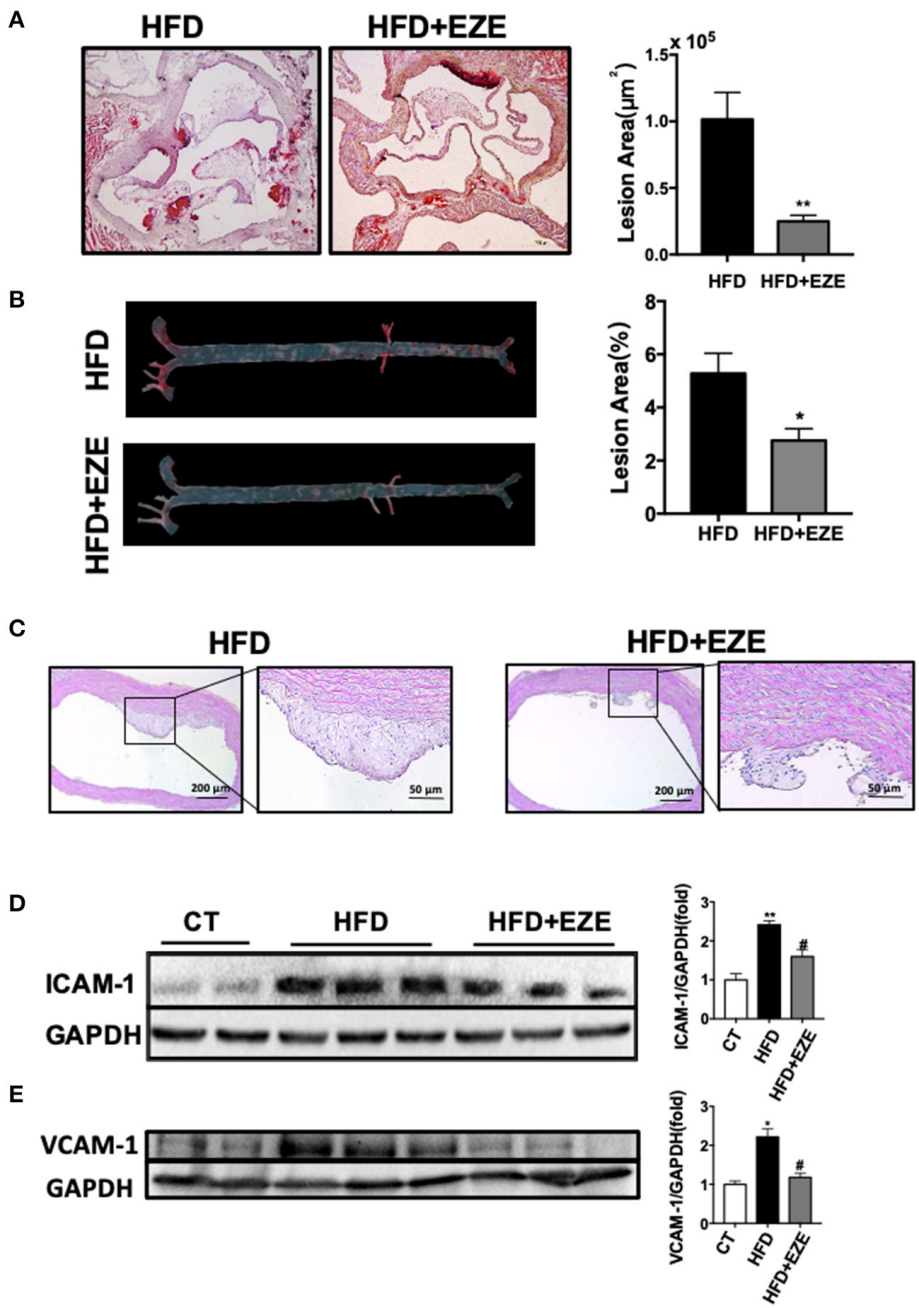
Atherosclerosis is attenuated in LDLR^−/−^ hamsters treated with ezetimibe after 40 days of HFD. Representative pictures of histochemistry sections of the aortic root **(A)** and *en face* analysis of the whole length of the aorta **(B)** by oil red O staining (*n* = 10). Scale bars, 500 μm. Total absolute oil red O positive area in all sections of the aortic root **(B)** and relative oil red O-positive area of the aorta *en face* (*n* = 10). Data are shown as mean ± SEM. ^*^p < 0.05, ^**^p < 0.01 compared with the HFD group, where the one-way ANOVA with Tukey's multiple comparisons test was performed. **(C)** Representative images of H&E staining of sections of the aortic arch. Scale bar = 200 or 50 μm, respectively. The expression of ICAM-1 **(D)** and VCAM-1 **(E)** in cultured HUVECs was analyzed by western blotting, the right panel was the quantitative bar chart analysis of the left panel. The serum from normal CD-fed WT hamsters was used as the control, the expression of ICAM-1 and VCAM-1 incubated with serum from HFD diet-fed hamsters was upregulated and downregulated with the serum from HFD-fed hamsters with ezetimibe administration. Data are shown as mean ± SEM. Two-tailed unpaired *t*-test, # HFD+EZE vs. HFD, ^#^*p* < 0.05, and the one-way ANOVA with Tukey's multiple comparisons test was performed.

The analysis of the pathology of the aortic root and the full length of the aorta *en-face* showed early-stage atherosclerotic lesions in LDLR^−/−^ hamsters (Figures 3A,B) and of note, this was not found in WT hamsters (Data not shown). Following ezetimibe administration, the lesion area decreased significantly by 75% at the aorta root and 50% at the full length of the aorta.

Sectioning of the aortic arch and staining with H&E (Figure 3C) found that fibrous cap structures have appeared in the plaques of HFD-induced hamsters, most of them were stable without necrotic cores, and also found the existence of unstable plaques in individual animals. In ezetimibe treated hamsters, the plaques are much reduced. Of note, ezetimibe can slow down the formation of atherosclerosis. However, when lesions from LDLR-/- hamsters with or without ezetimibe treatment were compared, no significant difference in inflammation or smooth muscle cell migration by immunohistochemistry staining was found (Supplementary Figure 4). In addition, when HUVECs were treated with serum from HFD-fed LDLR^−/−^ hamsters after ezetimibe was treated, ICAM-1 and VCAM-1 expression significantly reduced (Figures 3D,E).

Therefore, we believe that high concentrations of TRLs increased the susceptibility of atherosclerosis in the LDLR^−/−^ hamster which has high LDL levels. This may begin with an inflammatory response by endothelial cells in response to TRLs, and lipid deposition becomes severe with remnants and LDL uptake by macrophages.

### Severe Accumulation of Lipids in the Liver Was Significantly Reduced by Dietary Cholesterol Inhibition of Ezetimibe

**Figure 4:**
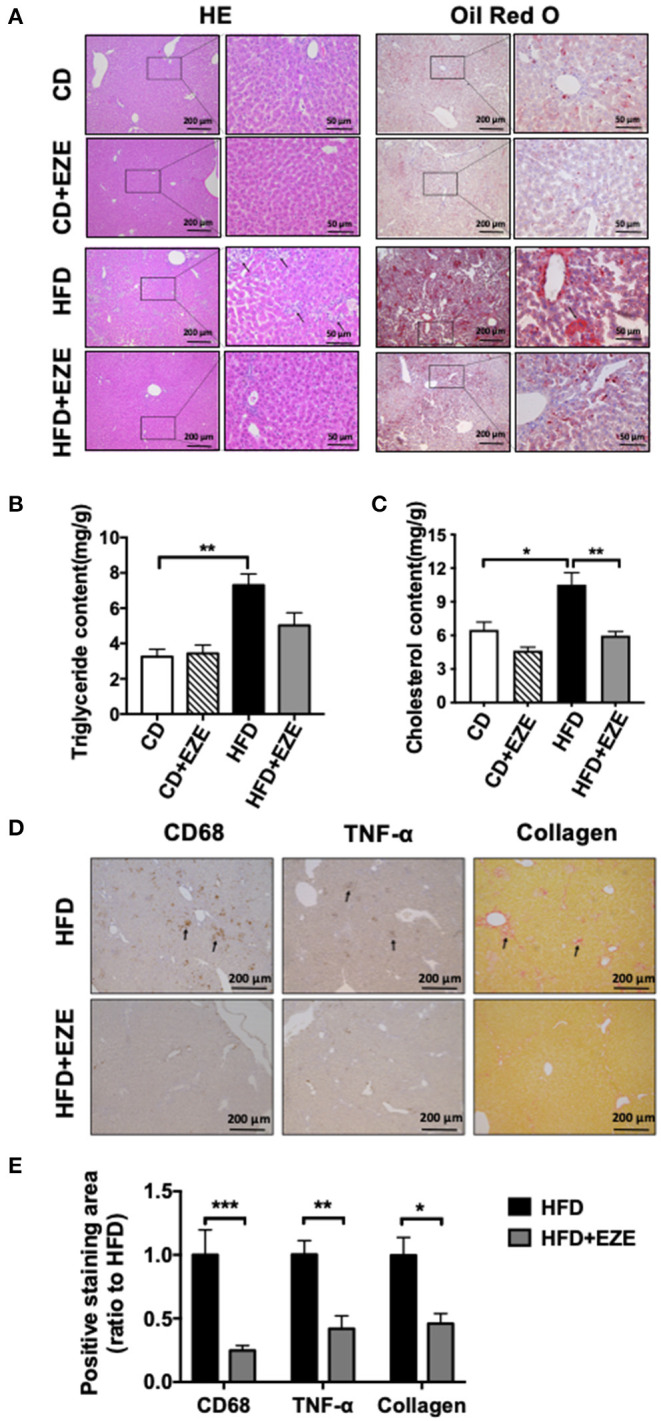
Hepatic morphological analysis and lipid extraction. LDLR^−/−^ hamsters were fed CD or HFD for 40 days and administrated with ezetimibe (+EZE) or solvent. **(A)** Representative images of liver cross-sections with H&E and oil red O staining. Scale bar = 200 or 50 μm, respectively. **(B)** Hepatic triglyceride content (*n* = 5), and **(C)** hepatic cholesterol content (*n* = 5). **(D)** Representative immunohistochemistry stained images of CD68 and TNF-α, and sirius red-stained images and **(E)** the relative quantization. Scale bar = 200 μm. Data are shown as mean ± SEM. Two-way ANOVA with Tukey's multiple comparison test, ^*^p < 0.05, ^**^p < 0.01, ^***^p < 0.001.

After 40 days of HFD, a severe accumulation of lipids in the livers of both LDLR^−/−^ and WT hamsters was found by H&E and oil red O staining, and upon lipid extraction. Ezetimibe treatment significantly reduced lipid deposition in the liver of both the LDLR^−/−^ (Figures 4A,B) and WT hamster (Supplementary Figure 4) induced by HFD-fed. Hepatic triglycerides were reduced by about 31% in the HFD-fed group treated with ezetimibe (Figure 4B). Hepatic cholesterol was reduced by about 30% in the CD-fed group and 45% in the HFD-fed group when treated with ezetimibe, respectively (Figure 4C). Unlike the plasma, lipid concentrations in the liver were not entirely reversed by ezetimibe, probably due to the upregulated synthesis of triglycerides but no significant increase of VLDL secretion (Supplementary Figure 2). Interestingly, H&E staining, along with anti-CD68 and anti-TNF-α immunohistochemistry staining, all showed an abundance of inflammatory cells in the liver of HFD-fed LDLR^−/−^ hamsters (Figures 4A,D). Sirius red staining showed that an HFD promoted fibrosis in the liver of LDLR^−/−^ hamsters. Ezetimibe attenuated both the inflammatory response and fibrosis (Figures 4D,E). In WT hamsters, the same results were found in the liver (Supplementary Figure 5E).

**Figure 5:**
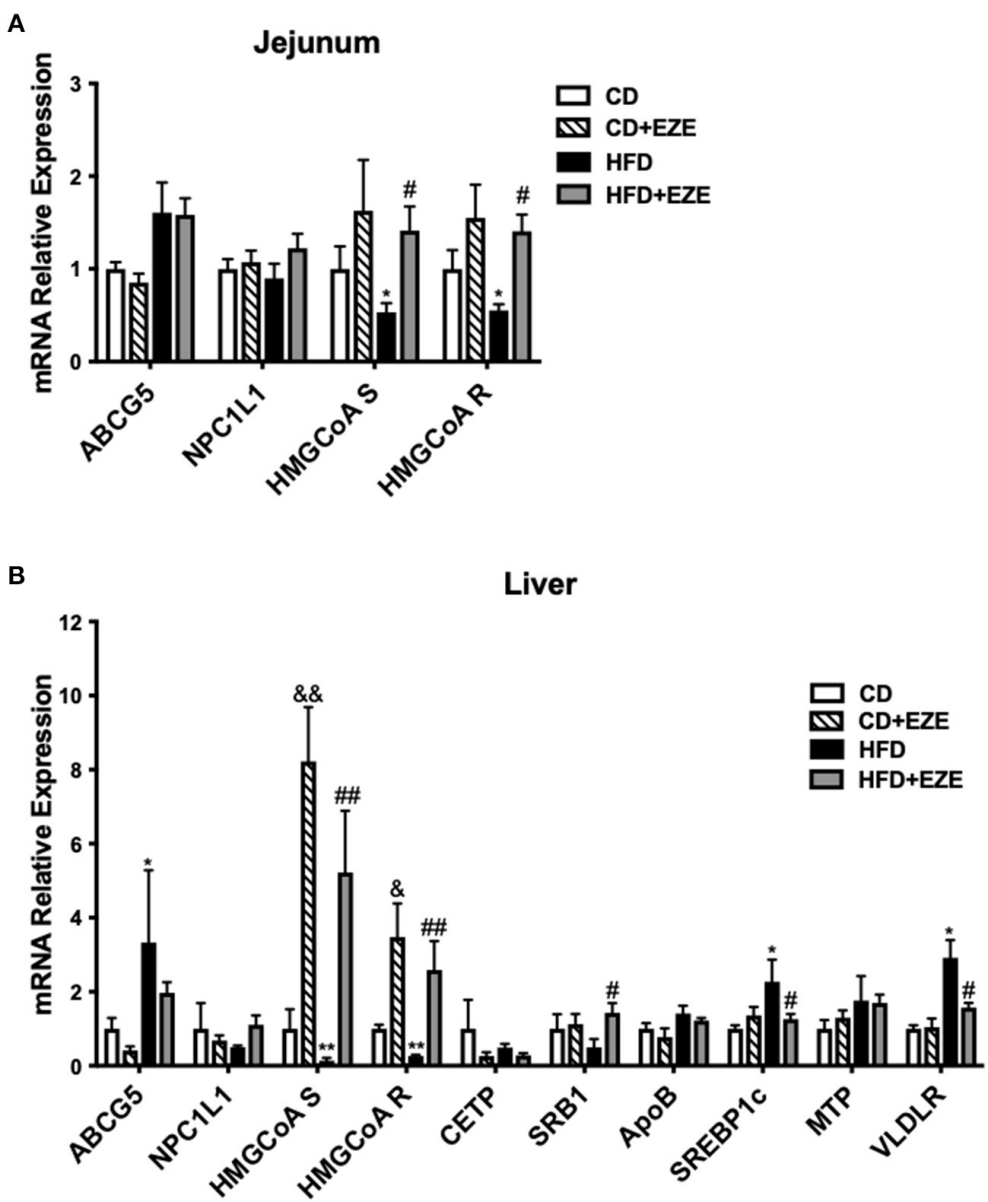
The expression of genes involved in lipid metabolism was determined by real-time PCR. LDLR^−/−^ hamsters were fed CD or HFD for 40 days with ezetimibe (+EZE) or solvent treatment. Total mRNA was extracted from the jejunum **(A)** or liver **(B)** for real-time PCR. Data are shown as expression relative to CD by mean ± SEM, *n* = 5. Two way ANOVA with Tukey's multiple comparison test, ^*^ HFD vs. CD, ^*^p < 0.05, ^**^p < 0.001; & CD + EZE vs. CD, ^&^*p* < 0.05, ^&&^*p* < 0.001; # HFD+EZE vs. HFD, ^#^*p* < 0.05, ^##^*p* < 0.001.

We quantified the expression levels of several major genes that are known to be involved in cholesterol (both intestinal and hepatic) and triglyceride metabolism and uptake in LDLR^−/−^ hamsters (Figure 5 and Supplementary Figure 6). Ezetimibe treatment significantly upregulated the expression of HMGCoA synthetase and reductase in both the liver and intestine, suggesting an increase in endogenous cholesterol synthesis. The expression of ABCG5 was upregulated in the liver and intestine in HFD-fed LDLR^−/−^ hamsters and ezetimibe treatment downregulated expression in the liver but not in the jejunum. SRB1 was downregulated by an HFD but was reversed in the liver upon ezetimibe treatment. NPC1L1 expression did not change in all groups. SREBP1c and VLDL receptor were both upregulated by HFD-fed LDLR^−/−^ hamsters and was attenuated by ezetimibe in the liver suggesting a response to dietary cholesterol. On the other hand, the genes of triglyceride synthesis and secretion were all no change during the CD but they were upregulated after HFD-fed, such as FAS and ACC1 in the liver and DGAT1, MTP, and ApoB in the intestine. Fas and ACC1, which are key enzymes that regulate fatty acid synthesis, were upregulated in the liver, suggesting that the synthesis of fatty acids in the liver was increased. DGAT1 and DGAT2, which are the key enzyme in the last step of synthesizing triglycerides. From the result, we found the expression of DGAT1 was increased in the jejunum, which suggested that the TG synthesis was increased in the jejunum. MTP is a key protein for the transfer of triglycerides into the blood, the increased expression of MTP indicated more triglycerides into the blood. FATP4 and ACSL5 have participated in triglycerides synthesis in the jejunum. CD36 is responsible for fatty acid intake. However, the expression of FATP4, ACSL5, and CD36 did not change after HFD-fed. The results suggested that accelerated synthesis of triglyceride but no change of secretion in the liver resulted in the fatty liver, and accelerated secretion in the intestine resulted in the plasma triglyceride increase.

## Discussion

This study described characteristics of the plasma lipid profile of a hyperlipidemia rodent model, the Syrian golden hamster, which was sensitive to, and regulated by, dietary lipids (11–14). Most significantly, in this model triglyceride levels were markedly increased by intestinally-derived cholesterol. The plasma lipid profile of the LDLR^−/−^ hamster after HFD-feeding was similar to that of postprandial hyperlipidemia in humans. Therefore, the LDLR^−/−^ hamster is a useful tool to study postprandial hyperlipidemia and the regulation of TRLs metabolism and associated diseases.

Our results showed that dietary lipids predominantly increased large particles of plasma lipoproteins in the Syrian golden hamster, especially in the LDLR^−/−^ hamster model. Unlike the ApoE knockout mouse (17), in these hamsters, the plasma lipid concentrations were elevated particularly high. These large lipoproteins contributed to increased plasma cholesterol and triglyceride levels by more than 80% in LDLR^−/−^ hamsters. The susceptibility of TRLs being elevated by an HFD is responsible for the particular increase of triglyceride in hamsters when compared with mice. But as with most rodents, the cholesterol level was also very high in TRLs.

The Syrian golden hamster has plasma CETP activity, and remarkably, this is a feature similar to human lipid metabolism. CETP has been reported to promote triglyceride synthesis and secretion by cells and impair triglyceride clearance in plasma (24–26). CETP activity significantly increased during the postprandial state, almost in parallel with an increase of plasma triglycerides (27, 28). A CETP inhibitor can increase triglycerides in TRLs, ApoE, and ApoC2 to promote the clearance of TRLs (29). Our data showed that ApoC3 and ApoA4 increased in the TRLs separated from HFD-fed LDLR^−/−^ hamsters. Therefore, it is possible that HFD-fed LDLR^−/−^ hamsters have increased TRLs and remnants, much like that in humans, due to CETP transferring HDL to VLDL and LDL, and changed apolipoproteins also may affect the characteristic of TRLs, thus delaying the clearance of triglycerides. We found that by knocking out the LDL receptor, there was a dramatic delay in the clearance of plasma triglycerides (Supplementary Figure 2). VLDL secretion did not change enough to affect blood lipid levels by diet, genotype, or ezetimibe (Supplementary Figure 2). The LDL receptor was also the receptor for lipoprotein remnants. Our data showed that expression of LRP1, another remnants receptor, did not change significantly in the liver (Supplementary Figure 6A). Extremely high levels of triglycerides in the LDLR^−/−^ hamster may largely be due to the delayed clearance of TRLs by LDL receptors. Therefore, compared with other rodents, hamsters have their distinguishing features in TRLs metabolism and further study needs to clarify the application of these characteristics into disease models.

Similar to humans, the editing of ApoB in hamsters happens only in the intestine (7), and upon apolipoprotein analysis, increases in ApoB48 and ApoE in plasma of HFD-fed LDLR^−/−^ hamster indicated an accumulation of chylomicron remnants. Through analysis of TRLs of CD-fed hamsters, it was also found that the levels of ApoB100/48 and ApoE in LDLR^−/−^ hamsters were significantly higher than in WT hamsters. Hence, our data also supported that chylomicron remnants accumulated when LDL receptor-deficient.

The study of Heek et al. (18) pointed out that ezetimibe alone may lead to a reduction in plasma cholesterol and triglycerides in humans with combined hyperlipidemia, such as obese insulin-resistant and/or patients with type 2 diabetes. In order to investigate the effect of dietary cholesterol, we used an NPC1L1 inhibitor, ezetimibe, to inhibit the absorption of cholesterol in HFD-fed animal models. Treatment with ezetimibe in LDLR^−/−^ hamsters resulted in a significant decrease in plasma triglycerides and TRLs. This showed that dietary cholesterol led to an accumulation of TRLs in hamsters, and this was mediated by LDL receptor deficiency. The study of Xia et al. (30) reported that ezetimibe may enhance RCT and expression of PPARγ to lower the lipids levels in high cholesterol high-fat diets. Additionally, we showed the effects on lipids levels in CD-fed LDLR^−/−^ hamsters. These results suggested that dietary cholesterol might play an important role in triglyceride metabolism in the Syrian golden hamster, a unique feature not seen in other rodents. Therefore, it will be helpful for understanding triglyceride metabolism and related diseases.

Unexpectedly, using FPLC analysis, it was found that HFD-fed LDLR^−/−^ hamsters had increased TRLs in the plasma only and ezetimibe only decreased these lipoprotein particles. LDL levels did not change in our experiments. Therefore, this feature can lend to the exploration of the effects of large lipoprotein particles and triglycerides. Analysis of FPLC data found that cholesterol accumulated in these large lipoprotein particles. We believe that these TRLs may be much more atherogenic. In this study, early-stage lesions, after 40-days of HFD, had reduced when ezetimibe inhibited TRLs but not LDL from the beginning. This is evidence of an important role of TRLs in the formation of initial lesions. Of note, these TRLs were mainly chylomicron remnants and relatively cholesterol-rich.

The study of Zilversmit proposed that increased chylomicrons/VLDL or their remnants in the postprandial plasma were major atherogenic lipoproteins (31). In the WT hamster, both TRLs and LDL were significantly increased after an HFD, and both contributed to the development of atherosclerosis. Thus, we are unable to delineate the roles of TRLs and LDL in this model. Based on genetically modified mouse models, such as LPL^−/−^, GPIHBP1^−/−^, and ApoC3 transgenic mice, triglycerides were demonstrated to promote atherosclerosis (5, 32–34). However, in these models, ApoB editing occurs in both the liver and intestine (35), along with the fact that they are insensitive to dietary lipids, which means that Apo48 levels in these models do not represent chylomicrons and their remnants, and one cannot distinguish postprandial lipoproteins. In LDLR^−/−^ hamsters, we offered clear evidence to support the association between TRLs and the formation of atherosclerotic lesions, as there was a significant increase of ApoB48 lipoproteins. These characteristics of the LDLR^−/−^ hamster provide a new experimental model to evaluate TRLs in the development of diseases in the context of atherosclerosis susceptibility.

Triglyceride-rich lipoproteins and their remnants activated inflammation and stress pathways, which in turn impaired endothelial function, leading to the infiltration of monocytes (35). Relatively small remnants in TRLs could also infiltrate into the artery wall like LDL (36). Subintimal lipoproteins may be modified and taken up *via* receptors on macrophages, such as SR-A, LRP1, the LDL receptor, the VLDL receptor, and so on, promoting the formation of foam cells and atherosclerotic lesions (37). These studies help us to understand hypertriglyceridemia as an independent risk factor for CVD. We also found that plasma with high TRL levels and remnants stimulated the upregulation of ICAM-1 and VCAM-1 when incubated with cultured HUVECs (Figures 3F,G). They activated the mitogen-activated protein kinase (MAPK) signaling molecules (32). Together, this suggested that atherosclerosis might begin with TRLs activating endothelial cells and was then promoted by increased monocyte adhesion and lipoprotein infiltration. Interestingly, we had found that macrophages from LDLR^−/−^ hamsters had a greater accumulation of lipids compared with WT hamsters (data not shown). Further, incubation of WT peritoneal macrophages with plasma from HFD-fed LDLR^−/−^ hamsters, which is rich in TRLs, resulted in an accumulation of lipids within these cells (data not shown). Whereas, TRLs incubated with LDLR^−/−^ hamster-derived macrophages also resulted in an accumulation of lipids within these macrophages. The hamsters can serve as an ideal model to investigate the effects of TRLs on diseases due to their TRLs levels can be manipulated (through HFD or ezetimibe). In other animal models, the effects of non-LDL lipoproteins on the development of atherosclerosis were not definitively excluded or properly investigated.

The liver plays an important role in lipid metabolism and fatty liver is usually associated with CVD. HFD-fed LDLR^−/−^ hamsters showed a significant accumulation of lipids in the liver. Interestingly, when ezetimibe inhibited the absorption of cholesterol, lipid deposition in the liver was also inhibited. This finding suggested that in hamsters, exogenous cholesterol plays an important role in both plasma and liver lipid metabolism and may be associated with atherosclerosis. The analysis of pathological sections also showed infiltration of inflammatory cells in liver tissue, suggesting that TRLs may induce inflammation in the liver, consistent with a previous report of liver inflammation in LDLR^−/−^ mice fed a cholesterol-rich diet (38).

Gene expression of cholesterol synthesis genes in the liver and intestine, such as HMG-CoA synthetize and reductase, were downregulated in HFD-fed LDLR^−/−^ hamsters and significantly upregulated after ezetimibe treatment. This reflects the effects of ezetimibe on cholesterol absorption and inhibition. Further, the finding was that ABCG5, a key gene for cholesterol efflux, was regulated by HFD and ezetimibe, which was also expected. The upregulation of Srebp1c and VLDLR after HFD-feeding may be associated with elevated TRLs. However, an understanding as to why SRB1 is downregulated is still unclear. LDLR^−/−^ mice did not show these changes (Supplementary Figure 7) related to triglyceride metabolism. The expressions of genes involved in triglyceride synthesis and secretion were not found to change in LDLR^−/−^ hamster in CD, suggesting that LDL receptor deficiency mainly caused the abnormal clearance of TRLs for elevated triglyceride. But these expressions upregulated in the liver or intestine after HFD, such as FAS, ACC1, MTP, ApoB, and DGAT1, indicated that the intestine derived lipoproteins and liver triglyceride accumulation in LDLR^−/−^ hamster after HFD fed.

In conclusion, the changes in lipoproteins in LDLR^−/−^ hamsters after HFD-feeding and ezetimibe treatment suggested that TRLs initiate and promote the factors involved in atherosclerosis. In addition, the LDL receptor may play an important role in triglyceride metabolism as the TRLs receptor. However, the patterns of lipid metabolism in hamsters need further investigation to understand its characteristics for better application. Although there are many aspects of the lipid metabolism of hamster more like humans, hamster also has many features, unlike humans, for example, extremely high lipids in plasma after HFD fed, a too easy rise of triglyceride, and maybe the difference of cholesterol ratio in lipoproteins. We hope that with the continuous application of hamsters in experiments, we can be more and more clear about which scientific problems are suitable to be solved with hamsters.

## Data Availability Statement

The original contributions presented in the study are included in the article/Supplementary Material, further inquiries can be directed to the corresponding author/s.

## Ethics Statement

The animal study was reviewed and approved by the Animal Care and Use Committee of the Peking University Health Science Center (LA2015-012).

## Author Contributions

XL performed most of the experiments. YW and XL wrote the original draft. PM, CY, JW, KH, and GC participated in the experiments and provided some data. WH, JF, and XX gave methodological conduct including molecular biology and pathology. YW and GL conceived the study and supervised the experiments. GL gave guidance in conceptualization and analysis. All authors contributed to the article and approved the submitted version.

## Funding

This study was financially supported by the National Natural Science Foundation (81770449 and 81570787) of China to YW and the National Key Research and Development Program of China from the Ministry of Science and Technology (2016YFE0126000) and Japan-China Sasakawa Medical Fellowship to YW.

## Conflict of Interest

The authors declare that the research was conducted in the absence of any commercial or financial relationships that could be construed as a potential conflict of interest.

## Publisher's Note

All claims expressed in this article are solely those of the authors and do not necessarily represent those of their affiliated organizations, or those of the publisher, the editors and the reviewers. Any product that may be evaluated in this article, or claim that may be made by its manufacturer, is not guaranteed or endorsed by the publisher.

## Abbreviations

α-SMA: α-smooth muscle actin
ABCG5: ATP-binding cassette transporter G5
ACC1: Acetyl-CoA carboxylase 1
ACSL5: Long-chain lipid coenzyme A synthase 5
ApoA1: apolipoprotein A1
ApoB: apolipoprotein B
ApoC3: apolipoprotein C3
ApoE: apolipoprotein E
BSA: bovine serum albumin
ECM: Endothelial Cell Medium
CD: chow diet
CD36: cluster of differentiation 36
CD86: cluster of differentiation 86
CETP: cholesteryl ester transfer protein
CM: chylomicrons
CVD: cardiovascular diseases
DGAT1: Diamide glycerin-based transferase 1
DGAT2: Diamide glycerin-based transferase 2
DTT: dithiothreitol
EZE: ezetimibe
FATP4: fatty acid transport protein4
FFA: free fatty acid
FPLC: fast protein liquid chromatography
GAPDH: glyceraldehyde-3-phosphate dehydrogenase
GPIHBP1: glycosylphosphatidylinositol-anchored high-density lipoprotein binding protein 1
FAS: Fatty acid synthase
H&E: hematoxylin-eosin staining
HDL: high-density lipoprotein
HFD: high cholesterol high-fat diet
HMG-CoA: 3-hydroxy-3-methyl-glutaryl coenzyme A
HUVEC: human umbilical vein endothelial cells
ICAM-1: intercellular cell adhesion molecule-1
VCAM-1: vascular cell adhesion molecule-1
LCAT: lecithin cholesterol acyltransferase
LDL: low-density lipoprotein
LDLR: low-density lipoprotein receptor
LPL: lipoprotein lipase
LRP-1: Lipoprotein receptor-related protein 1
mRNA: messenger ribonucleic acid
MTP: microsomal triglycerides transfer protein
NPC1L1: Niemann-Pick C1-Like 1
OCT: optimal cutting temperature
Ox-LDL: oxidative low-density lipoprotein
PBS: phosphate buffer saline
PCR: polymerase chain reaction
RIPA: radioimmunoprecipitation assay
RNA: ribonucleic acid
RPM: revolutions per minute
RT: reverse transcription
SDS: sodium dodecyl sulfate
SDS-PAGE: sodium dodecyl sulfate-polyacrylamide gel electrophoresis
SR-A: scavenger receptor A
SRB1: scavenger receptor B1
SREBP1c: sterol regulatory element-binding protein 1c
TNF: tumor necrosis factor
TRLs: triglyceride-rich lipoproteins
VCAM-1: vascular cell adhesion molecule-1
VLDL: very-low-density lipoprotein
VLDLR: very-low-density lipoprotein receptor
WT: wild type.
