# Supplementary material for: Dietary-Induced Elevations of Triglyceride-Rich Lipoproteins Promote Atherosclerosis in the Low-Density Lipoprotein Receptor Knockout Syrian Golden Hamster
Source: Front Cardiovasc Med. 2021 Nov 2;8:738060. doi: 10.3389/fcvm.2021.738060 (PMC8593475; doi:10.3389/fcvm.2021.738060)
Supplement: Supplementary file 1 [file Data_Sheet_1.docx]

**Supplementary Information**

**Supplementary material and methods**

**Oral Fat Load and VLDL Secretion Assay**

For the oral fat load assay, LDLR^-/-^ and WT hamster animals were fasted 12 hours, then gavaged with olive oil (10 mL/kg body weight). Plasma at the indicated time points (0 h, 0.5 h, 1 h, 2 h, 4 h, 6 h, 8 h and 12h) after gavage was collected for TG measurement. VLDL secretion was performed after 12-hour fasting of animals followed by intraperitoneal (i.p) injection with 7.5% Poloxamer 407 (Sigma, CAS 9003-11-6, 1500 mg/kg). Plasma was collected at 0, 0.5, 1, 1.5, 2, 3 and 4 hours after injection for TG measurement.

**Western blot analysis of TRLs fractions and SDS-PAGE for TRLs**

3 farctions of TRLs fraction(fraction 9-14) were pooled for apolipoprotein analysis. 15 μL of pooled samples were prepared with the buffer containing sodium dodecyl sulfate (SDS) and dithiothreitol (DTT) by heating at 95°C for 10 min. The samples were loaded to 6% SDS-PAGE gels and electrophoresed at 110 V. Proteins were transferred into nitrocellulose membrane for 180 min at 220 mA. Following blocking with 5% BSA, the membrane was hybridized with ApoB antibodies(ab20737, Abcam). The target bands were visualized by incubation with horseradish peroxidase conjugated secondary antibodies followed by enhanced chemiluminescence detection (Molecular Imager Gel Doc XR System, Bio-Rad, Hercules, CA, USA). As for SDS-PAGE of TRLs, 20 μg protein was loaded to 4%-15% gradient SDS-PAGE gels and electrophoresed at 150 V for 50 min.

**Supplementary Figures**

**Supplementary figure 1.** The effects of HCHF diet and ezetimibe on the lipid levels in wild type hamsters with normal chow diet for two weeks (A), and the effect of HCHF diet and ezetimibe on the lipid levels in wild type (B) or LDLR^-/-^ (C) hamsters with normal chow diet for 40 days used for atherosclerosis analysis. Atherosclerosis were induced with HCHF diet (HFD) for 40 days in LDLR^-/-^ (C, n=10) and WT (D, n=5) old hamsters. *p<0.05, ***p<0.001, one-way ANOVA with Dunnett’s multiple comparisons test was performed.


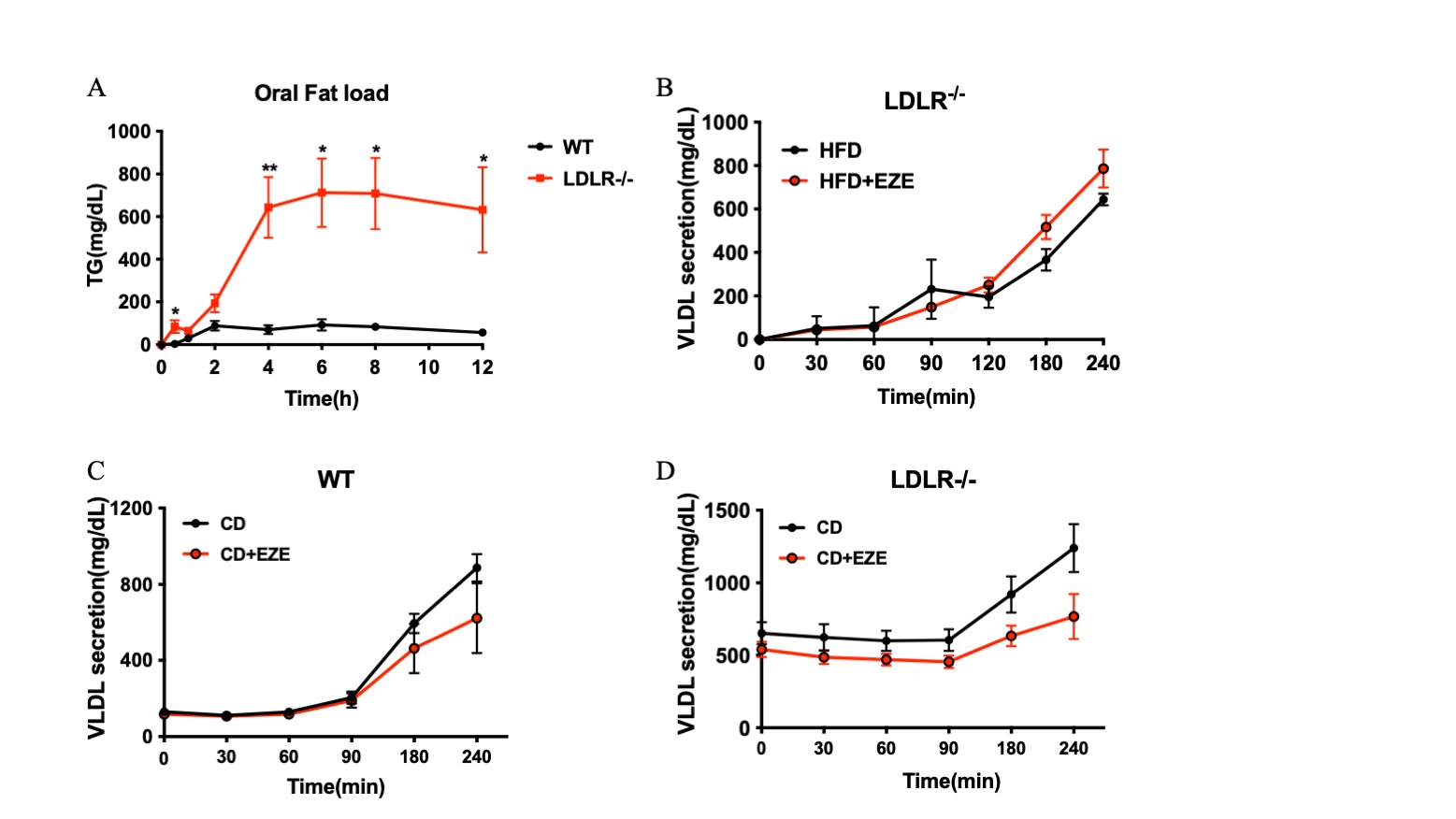


**Supplementary figure 2.** Oral Fat Load and VLDL secretion analysis of LDLR^-/-^ hamster and WT hamster. Data are shown as means ± SEM. Two-tailed unpaired t-test, *p<0.05, **p<0.01. A: Oral fat load test in wild type and LDLR^-/-^ hamsters (n=5) fed with chow diet. The result showed the delayed clearance of triglyceride in LDLR^-/-^ hamsters. B, C, D: VLDL secretion test was performed after intraperitoneal injection of P-407 (1500 mg/kg, n=5). The result showed that VLDL secretion was not affected by HFD in LDLR^-/-^ hamsters (B). In panel B the data is showed as increased TG to 0 min because the TG levels were too high after P-407 injection. When fed with chow diet ezetimibe may reduce the secretion of VLDL in hamster (C: wild type; D: LDLR-/-) although no significant differences were detected. There was no significant difference in VLDL secretion between wild type and LDLR^-/-^ hamsters.

**Supplementary figure 3.** Western blotting (A, B) for ApoB and ApoA5, ApoC3, ApoC2 were analyzed by SDS-PAGE (C) in plasma of LDLR^-/-^ hamsters with or without HFD and ezetimibe treatment after 2 weeks. (A) Western blotting for ApoB in density isolated TRLs and (B) in TRL fractions from the FPLC separation of LDLR^-/-^ hamsters. (C) SDS-PAGE analysis for ApoA5, ApoC3, ApoC2 in density isolated TRLs of LDLR-/- hamsters.


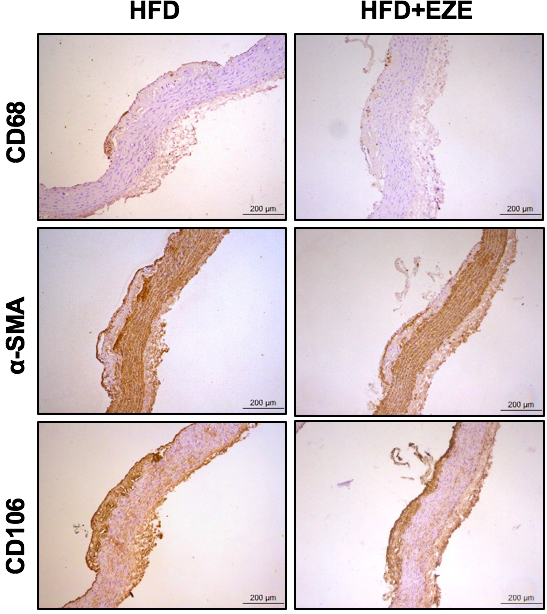


**Supplementary figure 4.** Representative images of immunohistochemistry staining of the same severe atherosclerotic plaques in aortic arch of LDLR^-/-^ hamsters with HCHF diet (HFD) feeding and ezetimibe (EZE) treatment by anti-CD68, α-SMA and CD106. Scale bar=200 μm. There are no significant different between the groups with ezetimibe treatment and the controls.


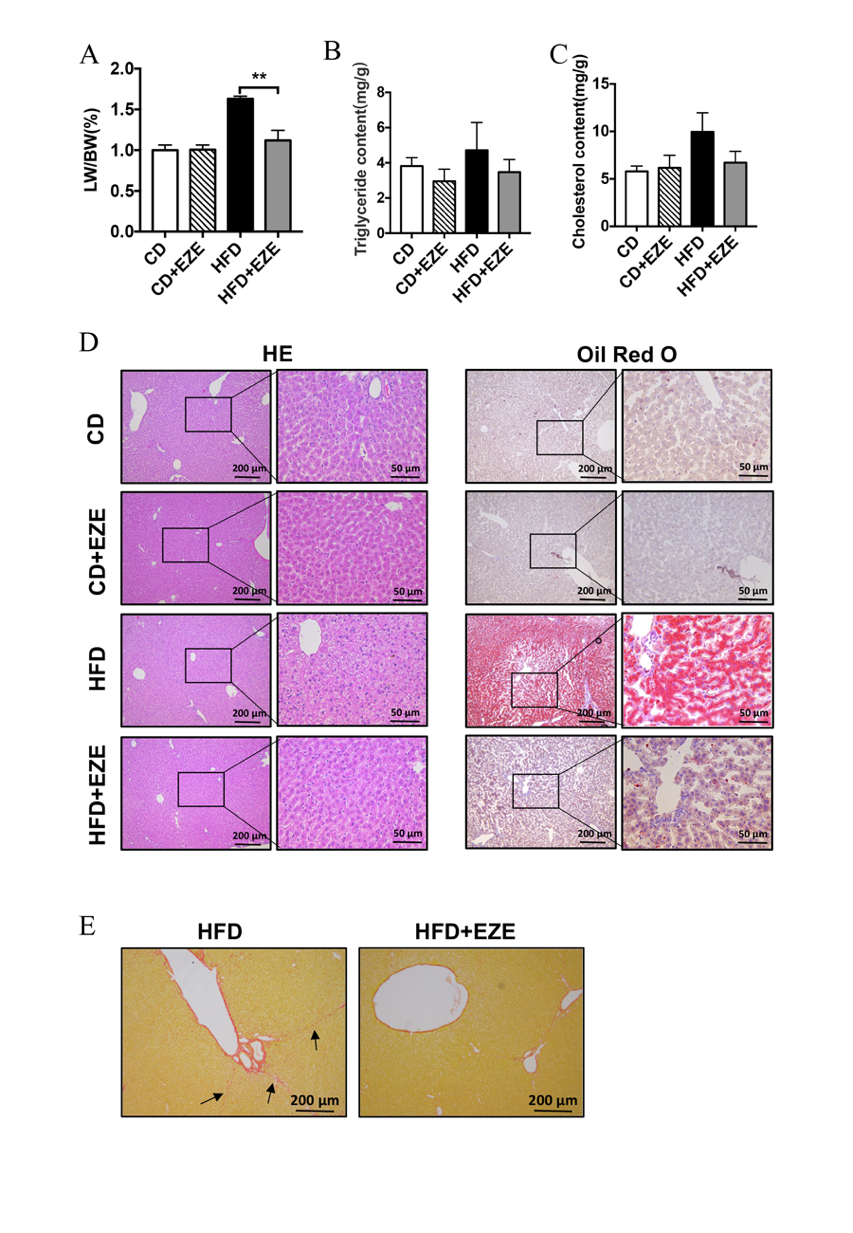
**Supplementary figure 5.** Effects of ezetimibe (EZE) on hepatic lipid levels and fibrosis in wild type (WT) hamsters. WT hamsters were fed chow diet (CD) or HCHF diet (HFD) for 40 days, ezetimibe reduced the accumulation of lipids in liver caused by HCHF diet as shown as the oil red O staining in panel D. A: The ratios of the liver to body by weights. B-C: Hepatic triglyceride and cholesterol extraction and determination. D: Representative images of H&E staining and oil red O staining by liver cross-sections. Scale bar=200 μm or 50 μm. E. Representative images of liver cross-sections with sirius-Red staining. Scale bar=200 μm. Data are shown as means ± SEM. Two-tailed unpaired t-test, **p<0.01, n=5.

**Supplementary figure 6.**The expressions of the genes involved in triglyceride uptake and metabolism in the jejunum, and triglyceride metabolism in the liver. LDLR^-/-^ hamsters were fed with chow diet (CD) or HCHF diet (HFD) for 14 days with ezetimibe (+EZE) or solvent treatment. Total mRNA was extracted from liver for RT-PCR. Data are shown as times to CD by means ± SEM, n=5. Two way ANOVA with Tukey’s multiple comparison test, * HFD vs CD，*p<0.05, **p<0.01; # HFD+EZE vs HFD, , ##p<0.01.

**Supplementary figure 7.** The expressions of the genes involved in lipid metabolism were determined by real-time PCR. LDLR^-/-^ mice were fed with chow diet (CD) or HCHF diet (HFD) for 14 days with ezetimibe (+EZE) or solvent treatment. Total mRNA was extracted from liver for RT-PCR. Data are shown as times to CD by means ± SEM, n=5. Two-tailed unpaired t-test, * HFD vs CD，*p<0.05; # HFD+EZE vs HFD, #p<0.05, ###p<0.001.

Primers list:

1. The primers used in real-time PCR of mouse.

VLDLRF: CAGCCCCAGCCACTCAC

VLDLRR: CAGCCCCAGCCACTCAC

MTPF: AAGAGGAGGAGCCTGTAGGG

MTPR: AGGCCTGTGGGTAGAAATGC

ApoBF: CGTGGGCTCCAGCATTCTA

ApoBR: TCACCAGTCATTTCTGCCTTTG

SR-B1F: TCCCCATGAACTGTTCTGTGAA

SR-B1R: TGCCCGATGCCCTTGA

SREBP-1cF: GGAGCCATGGATTGCACATT

SREBP -1cR: GGCCCGGGAAGTCACTGT

NPC1L1F: AGAACAAGACAGCAGGCTCC

NPC1L1R: CTTTTCTTTCCGGGCTTGGC

HMGCoASynF: GCCGTGAACTGGGTCGAA

HMGCoASynR: GCATATATAGCAATGTCTCCTGCAA

HMGCoARedF: CTTGTGGAATGCCTTGTGATTG

HMGCoARedR: AGCCGAAGCAGCACATGAT

GAPDHF: TGATGACATCAAGAAGGTGGTGAAG

GAPDHR: TCCTTGGAGGCCATGTAGGCCAT

2. The primers used in real-time PCR of hamster.

FASF: GCAGTCTTGAGTAGCTTTGTGCT

FASR: GGGAGCTGTCCAGATTAATACCT

ACC1F: ACACTGGCTGGCTGGACAG

ACC1R: CACACAACTCCCAACATGGTG

DGAT1F: TCTACCGGGACTGGTGGAAT

DGAT1R: CATCTGTTGCTGCCCAATCG

DGAT2F: GGGACAAGGATGTCCCACAG

DGAT2R: ACCCTTCCTCACACCAGACT

LRP1F: GGACCGTTCCGATGAGTCAG

LRP1R: GCTCAGGACAGGTCTTTTCGT

CD36F: TGTGTCTCCTTCAACGGTCA

CD36R: CCAGTTGCTCCACTCGTTTC

FATP4F: GTTTGCCTCAATGGTACGGC

FATP4R: CGGAAGGTCCAGTGAGTGTC

ACSL5F: GTTCCCAGTGGCTTCAAGTG

ACSL5F: GGCCTGTTGATCAGCCACAA
